# Editorial: New insights on vascular and metabolic diabetic complications

**DOI:** 10.3389/fphys.2026.1784609

**Published:** 2026-01-23

**Authors:** Hua Mao, John D. Imig, Ricardo Espinosa-Tanguma

**Affiliations:** 1 Department of Medicine, Baylor College of Medicine, Houston, United States; 2 University of Arkansas for Medical Sciences, Little Rock, United States; 3 Department of Physiology and Biophysics, School of Medicine, Universidad Autónoma de San Luis Potosí, San Luis Potosí, México

**Keywords:** diabetes, inflammatory, metabolism, metabolism–vascular crosstalk, vascular complications

## Introduction

Diabetes mellitus drives a global epidemic of vascular complications that span every level of the circulation–from microvascular injury in the brain, retina, kidney and heart to macrovascular atherosclerosis, stroke, and heart failure (Zakir et al., 2023; Li et al., 2023; Caturano et al., 2025; Mauricio et al., 2023). The 19 articles gathered in this Research Topic collectively underscore a central message: vascular disease in diabetes is not a simple downstream consequence of hyperglycemia, but the integrated outcome of metabolic, inflammatory, hemodynamic, and developmental insults acting across the life course. Together, these contributions map a path from molecular mechanisms to therapeutic strategies, illustrating how a more nuanced understanding of metabolism–vascular crosstalk can inform precision interventions.

## Metabolic timing, lipid signaling, and vascular injury

Various articles highlight that when and how metabolic pathways are perturbed can be as important as the magnitude of disturbance. Tian and Zhang dissect the interaction between feeding-fasting cycles, metabolic endotoxemia, and statin therapy, showing that simvastatin exerts opposing effects on high-density lipoprotein (HDL) cholesterol and glycemic control depending on metabolic state and Toll-like receptor 4 signaling. This work provides a conceptual framework for metabolic chronotherapy in cardiometabolic disease.

Post-translational lipid modification emerges as another key regulatory layer. Wang et al. review the role of protein S-acylation, also known as palmitoylation, in innate immunity, inflammation, and vascular injury across metabolic disorders, offering a unifying lens through which to interpret diverse cardiometabolic phenotypes. Their synthesis suggests that targeting palmitoylation machinery could address longstanding challenges related to specificity and off-target effects in drug development. Complementing this work, Liu et al. apply Mendelian randomization to interrogate causal relationships between polyunsaturated fatty acids and diabetic microvascular complications. Their genetic analyses indicated that higher omega-6 levels are associated with a lower risk of neuropathy and retinopathy in type 2 diabetes, whereas omega-3 fatty acids show no clear protective signal.

## Diabetic retinopathy and the blood–retinal barrier

The retina provides a sensitive window into diabetes-induced microvascular injury. Usenko et al. demonstrate marked upregulation of vascular endothelial growth factor (VEGF) and hypoxia-inducible factor-1α (HIF-1α) in experimental diabetic retinopathy and showed that combined insulin and sorafenib treatment normalizes VEGF levels and suppresses HIF-1α expression. Li et al. shift focus to mitochondrial quality control by reviewing mitophagy as a guardian of blood–retinal barrier integrity. They describe how high-glucose induced mitophagy dysfunction in endothelial cells, pericytes, and retinal pigment epithelium destabilizes both inner and outer blood–retinal barriers. Together, these contributions broaden the conceptualization of diabetic retinopathy from a purely angiogenic disorder to one that integrates hypoxia signaling, kinase cascades, and mitochondrial homeostasis.

## Heart and kidney: insulin signaling, oxidative stress, and microvascular dysfunction

The heart and kidney serve as archetypal target organs in diabetes, yet their pathophysiological trajectories are complex and intertwined. He et al. provides a comprehensive review of insulin signaling genes, including insulin receptor substrate (IRS)1, IRS2, phosphatidylinositol 3-kinase regulatory subunit (PIK3R1), and glucose transporter type 4 (GLUT4), and their roles in diabetic cardiomyopathy. By linking abnormalities in these pathways to impaired glucose transport, maladaptive remodeling, and heart failure, they frame insulin resistance as a gene-network disease within the myocardium rather than solely a systemic metabolic state. Furthermore, Bai et al. extend this mechanistic framework into therapeutics, demonstrating that the natural compound α-mangostin prevents diabetic cardiomyopathy in cellular and mouse models by attenuating oxidative stress and lipotoxicity. This integrated metabolic and oxidative axis provides a blueprint for multi-target cardioprotective strategies in diabetes.

At the level of renal microcirculation, Miller et al. identify the adaptor protein p66Shc as a contributor to diabetic nephropathy. Although structural kidney injury is modestly affected, this work reinforces p66Shc as a central integrator of oxidative stress and vascular dysfunction and highlights the importance of preserving microvascular reactivity before irreversible parenchymal damage occurs.

## Macrovascular disease, atherosclerosis, and valve calcification

Macrovascular disease remains the leading cause of mortality in diabetes, and multiple contributions address this challenge from complementary perspectives. Fang et al. report a controlled clinical trial showing that ginsenoside Rg1, when added to standard lifestyle and lipid-lowering therapy, reduces carotid plaque burden in older patients with diabetes while improving inflammatory markers, insulin resistance, blood pressure control, and cognitive performance. Xuan et al. identify clusterin as a key metabolic and immune regulator in diabetic atherosclerosis using gain- and loss-of-function murine models. Clusterin overexpression suppresses plaque formation, macrophage infiltration, inflammatory cytokine production, and pyroptotic cell death, whereas its deficiency exacerbates these pathological features, positioning clusterin as a promising therapeutic target.

Across the Research Topic, immune cells emerge as an active driver of diabetic vascular injury. As summarized by Ma et al., macrophages, T cells, and B cells contribute to both macrovascular and microvascular complications through sustained inflammatory signaling and immune imbalance. In large vessels, macrophage lipid uptake and foam cell formation amplify vascular inflammation, whereas in microvascular beds such as the kidney and retina, dysregulated T cell responses and cytokine signaling exacerbate endothelial dysfunction and barrier disruption. This synthesis highlights immune-related biomarkers, including soluble cytokine receptors and disease-associated microRNAs, as tools for early risk stratification and disease monitoring. At earlier disease stages, Wang et al. show that the systemic immune-inflammation index in humans with type 2 diabetes mediates the association between metabolic dysfunction-associated fatty liver disease and subclinical carotid atherosclerosis, reinforcing low-grade inflammation as a shared pathogenic driver.

Importantly, diabetic cardiovascular burden also extends beyond arteries to include calcific aortic valve disease. Liu and Cai review diabetes-associated calcific aortic valve disease as an active, regulable process in which inflammation, oxidative stress, and endothelial dysfunction link metabolic dysregulation to progressive valve fibrosis and calcification, with limited success to date for traditional lipid-lowering or renin-angiotensin system targeting in slowing disease progression. They further synthesize emerging evidence that glucose-lowering medications, including metformin, peroxisome proliferator–activated receptor γ (PPARγ) agonists, dipeptidyl peptidase-4 (DPP-4) inhibitors, glucagon-like peptide-1 (GLP-1) receptor agonists, and sodium-glucose co-transporter 2 (SGLT-2) inhibitors, may offer pharmacologic leverage to retard calcific aortic valve disease through metabolic, anti-inflammatory, and anti-calcific mechanisms, complementing their established cardiovascular benefits.

## Vascular dysfunction before and beyond diabetes

A recurrent theme is that vascular injury often precedes overt diabetes and extends beyond traditional cardiovascular endpoints. Romero-García et al. review how insulin resistance and altered calcium handling in vascular smooth muscle cells contribute to early vascular dysfunction in prediabetes and metabolic syndrome. They emphasize a functional unit composed of voltage-gated L-type CaV1.2 calcium channels, sarcoplasmic/endoplasmic reticulum calcium ATPase (SERCA) pumps, ryanodine receptors, and large-conductance calcium-activated potassium (BK_Ca_) channels that regulate resistance artery tone and vasorelaxation. Alejandra et al. extend the temporal perspective to gestation, synthesizing evidence that maternal hyperglycemia and metabolic disturbances program offspring for future hypertension and cardiovascular disease. Shared mechanisms include oxidative stress, endoplasmic reticulum stress, nitric oxide deficiency, and structural arterial remodeling.

The brain represents another vulnerable target. Venuti et al. link metabolic biomarkers to recognition memory deficits and hippocampal pathology in diabetic mice, showing that partial restoration of normoglycemia improves both cognitive performance and tissue structure. Zhu et al. focus on acute cerebrovascular disease, demonstrating that the stress hyperglycemia ratio in humans is strongly and non-linearly associated with mortality after hemorrhagic stroke, highlighting acute glycemic stress as a critical prognostic factor.

## Exercise, microcirculation, and integrative physiology

Several contributions address the role of microcirculation and exercise physiology in type 2 diabetes. Russell McEvoy et al. show that insulin-mediated increases in red blood cell velocity, oxygen delivery, and capillary recruitment are blunted in diabetic rats, even under controlled metabolic conditions. Madsen et al. review cardiovascular determinants of reduced exercise cardiac output in patients with type 2 diabetes, including myocardial remodeling, coronary microvascular dysfunction, and skeletal muscle rarefaction. Together, these studies emphasize that restoring metabolic control without improving microvascular adaptability leaves substantial functional impairment unaddressed.

## Converging themes and future directions

This Research Topic highlights a wide spectrum of therapeutic strategies, including small molecules, phytochemicals, endogenous proteins, lifestyle timing, and lipid composition. Multiple unifying principles emerge targeted modulation rather than blanket suppression of stress pathways; inflammation and immunity as central hubs linking metabolism and vascular injury; the importance of temporal and developmental context; and microvascular function as a sensitive and actionable endpoint. Future progress will require cross-disciplinary approaches that integrate genetics, mechanistic biology, functional imaging, and clinical stratification beyond glycemic indices alone.

## Conclusion

Collectively, these studies advance our understanding of how diabetes and related metabolic disturbances drive vascular and organ damage while illuminating a broad landscape of therapeutic opportunities. By continuing to bridge molecular mechanisms, integrative physiology, and clinical translation, the field is well positioned to move beyond glucose-centric care toward genuinely vascular-protective and organ-protective strategies for people living with diabetes.
